# The Effects of High-intensity Functional Training (HIFT) on Spatial Learning, Visual Pattern Separation and Attention Span in Adolescents

**DOI:** 10.3389/fnbeh.2020.577390

**Published:** 2020-09-14

**Authors:** Tavor Ben-Zeev, Tamir Hirsh, Inbal Weiss, Michael Gornstein, Eitan Okun

**Affiliations:** ^1^The Mina and Everard Goodman Faculty of Life Sciences, Bar-Ilan University, Ramat Gan, Israel; ^2^The Leslie and Susan Gonda Multidisciplinary Brain Research Center, Bar-Ilan University, Ramat Gan, Israel; ^3^The Paul Feder Laboratory on Alzheimer’s Disease Research, Bar-Ilan University, Ramat Gan, Israel; ^4^Herzliya Municipality, Herzliya, Israel

**Keywords:** cognition, adolescents, spatial learning, pattern separation, high-intensity functional training, attention

## Abstract

Aerobic, anaerobic, and strength exercises are known to improve various cognitive functions, such as executive functions, pattern separation, and working memory. High-intensity functional training (HIFT) is a form of physical activity that can be modified to any fitness level and elicits greater muscle recruitment than repetitive aerobic exercises, thereby improving cardiovascular endurance, strength, and flexibility. HIFT emphasizes functional, multi-joint movements *via* high-intensity interval training (HIIT) and muscle-strengthening exercises. It is yet unknown, however, whether HIFT affects cognitive functions in adolescents. To address this question, we subjected adolescents to 3 × 20 min training sessions/week of HIFT for 3 months. The effects of HIFT were tested on performance in: (1) virtual reality (VR)-based spatial learning task; (2) computerized visual pattern separation; and (3) attention span. The control group performed a typical physical class three times per week. The effects on cognition were tested at baseline and following 3 months of HIFT. Three months into the intervention, the HIFT group achieved higher scores in the spatial learning task, pattern separation task, and in the attention span test, compared with controls. These data suggest that HIFT can potentially translate into improving school performance in adolescents.

## Introduction

It is well established that aerobic exercise improves various cognitive functions in humans, such as executive functions, pattern separation, and neuronal plasticity (Ploughman, 2008; Holzschneider et al., 2012; Barak et al., 2015). An even greater range of cognitive domains was found to be affected by exercise in studies involving rodents (Aguiar et al., 2011; Lee et al., 2012). Findings in human studies are consistent with research in rodents, suggesting that physical activity may provide lasting benefits for brain structure and function (Voss et al., 2013). For example, aerobic exercise improves hippocampal memory tasks such as visuospatial memory for relationships between landmarks on maps (Woost et al., 2018). At present, it is believed that the mammalian brain exhibits persistent plasticity throughout all stages of life (Déry et al., 2015). Neuronal plasticity allows the central nervous system (CNS) to learn new skills, to consolidate and retrieve memories, to reorganize neuronal networks in response to environmental stimuli, and to recover after lesions. It is well established that physical exercise promotes the proliferation of new neurons within the dentate gyrus (DG) of the hippocampus in animal models, namely, adult hippocampal neurogenesis (Van Der Borght et al., 2007; Lee et al., 2012). It is thought that hippocampal neurogenesis contributes to several aspects of cognitive learning processes, including pattern separation, i.e., the ability to differentiate between highly similar sensory inputs (Hartley et al., 2014).

High-intensity functional training (HIFT) is a training program that incorporates a variety of multi-joint functional movements performed at high-intensity and designed to improve parameters of general physical fitness (cardiovascular endurance, strength, and body composition) and performance (agility, speed, power, and strength; Buckley et al., 2015; Heinrich et al., 2015; Haddock et al., 2016; Feito et al., 2018a). HIFT utilizes both high intensity “anaerobic exercise” (e.g., jumping, sprinting) and muscle-strengthening exercises (e.g., push-ups, squats; Heinrich et al., 2015; Haddock et al., 2016; Feito et al., 2018a). Also, HIFT has a greater muscle recruitment factor compared with “aerobic exercises,” having the ability to improve cardiovascular endurance and strength (Buckley et al., 2015; Feito et al., 2018a).

HIFT has been shown to improve maximal oxygen consumption (Heinrich et al., 2012, 2015), decrease body fat (Feito et al., 2018b), as well as showing improvements in bone mineral content (Feito et al., 2018b). Participants subjected to 8 weeks of HIFT improved muscle strength performance on the bench-press and leg-press exercises, as well as their muscular aerobic performance on 1-min sit-up and sit-and-reach tasks (Brisebois et al., 2018). Also, after 8 weeks of HIFT, subjects improved their absolute VO2max and relative VO2max (Brisebois et al., 2018). These data show that not only does HIFT improve aerobic and anaerobic abilities, it also improves muscular strength performance. Another key aspect of HIFT for the general population is that individuals who perform HIFT reported higher levels of motivation measured using the “exercise motivations inventory-2” (EMI2) questionnaire that consists of six point scale from 0 (not at all true for me) to 5 (very true for me). The HIFT group exhibited higher motivation compared with strength training group (HIFT; 4.5 vs. strength training; 3.5, EMI2 score; Heinrich et al., 2014; Fisher et al., 2017).

HIFT is not the only training paradigm that provides cognitive benefits with a relatively short-time requirement. Of note, high-intensity interval training (HIIT) has a significant effect on cognitive abilities in humans and rodents alike (Afzalpour et al., 2015; Tsukamoto et al., 2016; Fiorelli et al., 2019). HIIT refers to an exercise paradigm that uses short bursts of aerobic activity, interspersed by periods of rest or low-intensity exercise for recovery (Gibala et al., 2012). Although HIFT and HIIT share some similarities they are distinct in the following: HIIT utilizes only aerobic exercises performed at very high intensity with no variation (Gibala et al., 2012; Calverley et al., 2020). HIFT, on the other hand, utilizes a varied muscle-strengthening and high intensity “anaerobic” exercises (Feito et al., 2018a). While both training programs, HIFT and HIIT, significantly improve aerobic capacity (7 and 5%, respectively), and anaerobic power (15 and 12%, respectively); After 6 weeks of HIFT training, muscle strength, power, and muscular endurance were significantly improved compared with HIIT (Buckley et al., 2015). This is an indication that HIFT is a valid and more effective training paradigm compared with HIIT in improving muscle strength. Strength training was shown to improve various cognitive abilities, like working memory and executive function (Cassilhas et al., 2007; Gibala et al., 2012; Fortes et al., 2018; Herold et al., 2019; Landrigan et al., 2019). It was also shown to increase the levels of brain-derived neurotrophic factor (BDNF), an important factor for neuronal plasticity and survival (Quiles et al., 2019; Mckay et al., 2020). Strength training can be described as a training program that aims to improve the individual ability to produce force, increasing muscle mass (American College of Sports Medicine, 2009). Skeletal muscle, the largest organ in the human body (Pedersen and Febbraio, 2012), systemically secretes myokines, such as BDNF, IRISIN, and Insulin-growth factor-1 (IGF-1), which are important for cognition. These pleiotropic factors promote neurogenesis, neuronal survival and neuroplasticity (Pedersen and Febbraio, 2012; Jin et al., 2018; Lourenco et al., 2019; Martínez Muñoz et al., 2019), and cognitive abilities (Cotman et al., 2007; Lee et al., 2012). Several studies have shown that strength training increase BDNF and IGF-1 (Hess and Smart, 2017; Kelty et al., 2019; Quiles et al., 2019; Mckay et al., 2020), more so than aerobic exercise in the CNS, shown by studies in rodents, and in the circulation, shown in studies in humans (De Souza Vale et al., 2009; Chen et al., 2017; Hess and Smart, 2017; Kelty et al., 2019). Strength training and HIIT were both shown to exert positive effects on various types of cognitive abilities (Cassilhas et al., 2007; Tsukamoto et al., 2016; Fiorelli et al., 2019; Landrigan et al., 2019). As a result, we aimed to investigate how a training paradigm that combines both strength exercise and high-intensity aerobic exercise affects different cognitive domains.

Spatial learning is the process in which information about the environment is encoded to allow navigation through space and recall the location of motivationally relevant stimuli (Tan et al., 2017). This form of learning is critically dependent on the integrity of the hippocampus (Witter et al., 2000) and parahippocampal regions, such as the medial entorhinal cortex (MEC; Moser et al., 2008). Concerning humans, the theory also suggests lateralization of hippocampal function, with the right hippocampus encoding spatial relationships and the left hippocampus storing relationships between linguistic entities (Iglói et al., 2010). Moreover, one or both hippocampi incorporate temporal information derived from the frontal lobes, which serves to timestamp each visit to a location, thus providing the basis for a spatial short-term working memory system (which can hold information from seconds and up to several minutes; Burgess et al., 2002). Results from human neuropsychology, neuroimaging, and electrophysiology experiments strongly suggest an evolutionary continuity spanning mammalian species and implicating the hippocampal formation and its cortical inputs in allocentric spatial processing in rodents, primates, and humans alike (Banta Lavenex et al., 2014; Hartley et al., 2014). Most of the computer-based tasks that assess spatial learning do not involve the hippocampus or the EC, as in the case of the dots fixation task (Nagamatsu et al., 2013) and the 3D rotational tasks (Holzschneider et al., 2012), suggesting the improper use of tools to assess spatial learning in humans. In contrast, virtual reality (VR) environments have been used to assess spatial learning and memory in humans (Snider et al., 2013), due to their capability to activate the cell assembly involving in spatial learning as suggested in rodents, thus enabling to mimic real-world navigation. Because of these problems, we utilized VR goggles, for which we developed spatial cognition tasks. This enabled us to assess the long-term and short-term spatial learning and memory capacity of human participants by examining their ability to return to the exact location of a fixed target goal in a large environment with extra-maze cues. The VR task is an analog task to the rodent’s most widely used tests for assessing short-term spatial cognition abilities, namely, the Morris water maze (MWM). Several studies have shown the positive effect of different types of exercise on spatial learning in humans and rodents alike (Holzschneider et al., 2012; Lee et al., 2012; Nagamatsu et al., 2013; Barak et al., 2015; Cassilhas et al., 2016).

Pattern separation was previously shown to be affected by voluntary aerobic exercise in rodents, as measured using the Bussey touch-screen system (Creer et al., 2010; Bekinschtein et al., 2011; Cassilhas et al., 2016). In humans, pattern separation, as measured using Mnemonic Similarity Task (MST) and putative neurogenesis-dependent task, was also affected by moderate aerobic exercise (Déry et al., 2013; Bernstein and Mcnally, 2019). Pattern separation, an important part of episodic memory, is the ability to identify similar inputs into distinct, non-overlapping new memories that can be stored rapidly without inducing large amounts of interference (Mcclelland et al., 1995; Norman and O’reilly, 2003). Studies investigating pattern separation have shown that during pattern separation tasks, the CA3 region in the brain is critical for associative memory formation and retrieval of memories (Gilbert and Kesner, 2003). Serval studies showed that DG has an important role in resolving interference between highly similar contexts (Rolls and Kesner, 2006; Mchugh et al., 2007; Hunsaker et al., 2008). Some studies have shown that exercise improves pattern separation abilities in rodents (Creer et al., 2010; Bekinschtein et al., 2011; Bernstein and Mcnally, 2019), and one study showed that HIIT in adult men increases performance in a pattern separation task (Heisz et al., 2017). In the current study, we used the MST task to assess pattern separation abilities. This task is specifically designed to test pattern separation and has been shown to successfully assess differences and improvements in pattern separation abilities (Stark et al., 2013; Clemenson et al., 2020).

Inhibitory control, the suppression of behavior in response to either internal or external influences (Fuster, 1997), is a cognitive function important to many everyday situations (Coxon et al., 2007). These include controlling our self-regulation of instinctive responses that assists in selecting, scheduling, and coordinating the processes that underlie perception, memory, and action (Passingham, 1996). The prefrontal cortex (PFC) is a brain region that plays a key role in inhibitory control tasks (Miller and Cohen, 2001; Munakata et al., 2011). Many studies have shown the positive effect of exercise on inhibitory control abilities (Browne et al., 2016; Peruyero et al., 2017; Tsuk et al., 2019), with one study showing a positive effect for a high-volume resistance training program on inhibitory control abilities (Fortes et al., 2018). In the current study, we have assessed the effects of HIFT on inhibitory control abilities, using the Stroop test that has been used widely to assess inhibitory control abilities (West and Alain, 2000; Zysset et al., 2001; Adams and Jarrold, 2009).

In the current study, we examined how 3 months of HIFT program affects spatial learning using VR, pattern separation, and inhibitory control on adolescents/preadolescents. Because HIFT is less time consuming and has more adherence compared with more traditional types of training we hypothesized that this type of training is more suitable for adolescence (Heinrich et al., 2014; Fisher et al., 2017; Feito et al., 2018a). Adolescents experience significant changes in hormonal levels (Reiter and Root, 1975). Concerning growth and development, testosterone contributes the most and is significantly elevated in adolescents (Nottelmann et al., 1987; Griggs et al., 1989). Testosterone was shown to have a robust positive effect on cognitive function and brain development (Beauchet, 2006; Nguyen et al., 2013; Hua et al., 2016). It is well established that strength training significantly increases testosterone levels (Spiering et al., 2009; Vingren et al., 2010; Hooper et al., 2017). Since HIFT includes strength exercise, we hypothesized that this type of training can have a greater positive effect on cognition in adolescence. This takes into consideration their already increasing levels of testosterone and the fact the strength exercise increases testosterone levels in adolescence (Zakas et al., 1994).

## Materials and Methods

### Ethical Approval

The study was approved by Bar-Ilan University’s Ethics Committee following the department of Research at the Israeli Ministry of Education.

### Participant Inclusion Criteria

We recruited 40 male subjects (*n* = 20 in each group; control and experimental) aged 12–13 from two adjacent middle schools. Students in the HIFT and control groups were recruited and trained in two separate middle schools. Both middle schools represent the same socioeconomic status exhibit identical education program. Calculating the required sample size for a given effect size, indicated that 40 participants will be required for each gender. Recruiting both males and females was not feasible within the current study. Before the beginning of the study, participants’ parents were required to sign informed consent forms, which were approved by the department of Research at the Ministry of Education. Inclusion criteria required that participants were not taking medication for the treatment of mental disorders or cognitive enhancers before and during the study. Individuals diagnosed with ADHD were excluded from the study. Participants who failed to attend at least 90% of the training program were excluded from the study.

### Experimental Design

The intervention group (*n* = 20) performed a short HIFT program three times per week, while the control group performed the regular physical exercise class (moderate intensity) three times per week. Before the 3-month intervention, cognitive tests were conducted at baseline as well as at the 3-month endpoint.

### Training Program

**Figure 1:**
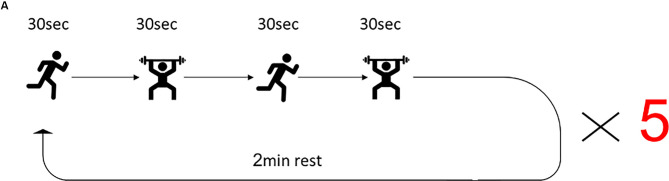
High-intensity functional training (HIFT) program. The training session consisted of 30 s high-intensity aerobic exercise, followed by 30 s strength exercise, followed by 30 s different high-intensity aerobic exercise, followed by different 30 s strength exercises. This sequence of exercises was repeated five times for a total of 20 min training session and was conducted three times/week.

The training program for the HIFT group was guided by a certified instructor. The control group performed the regular physical exercise classes led by the schoolteacher that consist of 45 min moderate-intensity cardiovascular exercises and included jogging and sports games such as soccer and basketball. The HIFT program included a 20 min combination of multi-joint functional movements performed at high intensity. Before the HIFT session, participants conducted a 10 min dynamic warmup. The HIFT program consisted of the 30 s of high-intensity “anaerobic exercise” (e.g., sprints, jumps) paired with 30 s of body weight/free-weight resistance exercise (e.g., pushups, squats, presses), and again 30 s of highly intense “anerobic exercise” paired with 30 s of body weight/free-weight resistance exercise, which started with bodyweight only and incrementally increase the difficulty by including free weights. Upon completion of one round of exercise, subjects received a 2-min rest, followed by four repeats of this cycle, to a total of five cycles (Figure 1). Following the completion of the session, subjects performed a cool-down set that consists of 5 min of stretching. The exercise was conducted three times a week, every Tuesday, Thursday, and Friday, for the duration of the study.

### Cognitive Assessment

Herein, we tested whether 3 months of HIFT training affected three different cognitive domains, namely, spatial learning, pattern separation, and inhibitory control in adolescents, as follows:

#### Spatial Learning

**Figure 2:**
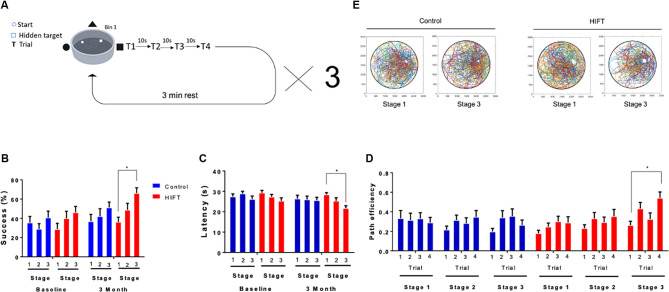
HIFT enhances performance in the VMWM. Participants (*n* = 40) were randomized into intervention or control group (*n* = 20/group). The intervention group followed a HIFT program that consisted of three sessions/week for 20 min. The control group followed the standard physical class that consisted of three sessions/week for 45 min. **(A)** Before conducting the training program, participants preformed a virtual reality (VR) test to examine the baseline spatial learning abilities. Participants were tested again at the end of the training program in the same spatial learning VMWM task. The following parameters were measured: **(B)** success rates to find the target, **(C)** latency to reach the target and success rates to find the target, **(D)** path efficiency by trials, and **(E)** movement plots (*p < 0.05).

Short-term spatial memory was tested using VR variant of the “MWM.” The subject’s success rates to find the target, latency to reach the target and path efficiency (Euclidean distance between the starting point and the final point divided by the total distance) were measured. The task consisted of a black and white arena (width: 51 m × length: 51 m × height: 27 m), with three different cues on different walls and a dark circular area in it (diameter = 25 m). The task was divided into three stages separated by 3 min inter-trial-intervals. Each stage included 4 × 35 s trials with three cues on the walls to find the hidden target zone (Figure 2A). If the subject found the target zone within the 35 s time frame, an appropriate text box appeared on the screen. Otherwise, the subject was transferred to the desired location while being informed about it. When reaching the location of the platform, subjects had 10 s to observe the extra maze cues and memorize the platform’s location. At the end of the 10 s, subjects started the next trial at a different location. At the end of the last trial, the subject was informed that the test was done. After finishing the first stage, a 3 min rest period was given to the subjects, following which they performed stages 2 and 3. Before the beginning of the first stage, the subjects performed a familiarization phase that included three trials to find a visible red rectangular target zone in the VMWM. This phase is important for habituation to the VR goggles as well as for navigation with the controller (X-Box, Microsoft). It also allows the participants to understand better the task due to its similarity to the test stage. The platform location in all three stages did not change between the baseline test and the 3 months test.

#### Pattern Separation

**Figure 3:**
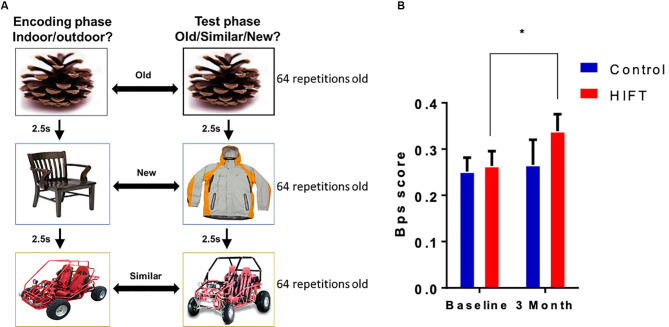
HIFT enhances performance in the Mnemonic Similarity Task (MST) task. **(A)** The participants performed the MST task before conducting the training program, to assess baseline pattern separation abilities. Participants were tested again at the end of the training program in the same MST task. To assess pattern separation, we measured. **(B)** Lure discrimination index (LDI) score was defined as the number of “similar” responses given to “lure” items minus the number of “similar” responses given to “foil” items (*p < 0.05).

The MST task consisted of a series of 192 color photographs of everyday objects on a white background. In the first phase, participants engaged in an indoor judgment for each picture *via* a button-press (128 items total, 2 s each, 0.5 s interval between pictures). Immediately following the encoding task, participants were given instructions regarding a surprise recognition memory test in which they identified each item as “Old”, “Similar”, or “New” *via* button-press (192 items total—64 repeated items, 64 lure items, and 64 foil items; 2 s each, 0.5 s interval between pictures). One-third of the images in the test phase were exact repetitions of images presented in the study phase (targets); one-third of the images were new images not previously seen (foils), and one-third of the images were similar to those seen during the study phase, but not identical (lures; Figure 3A). Behavioral pattern separation performance indicated by the “lure discrimination index (LDI),” was calculated as the difference between the rate of “Similar” responses given to the lure items minus “Similar” responses given to the foils items (to correct for response biases). Besides, we also measured a “recognition score” calculated as the difference between the rate of “Target” responses given to the old items minus “New” responses given to the old items. The images used in the MST task were identical for the baseline and the 3-month time point.

#### Inhibitory Control

**Figure 4:**
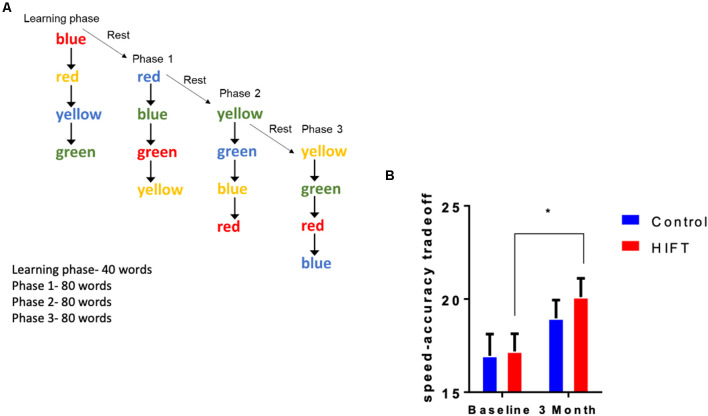
HIFT enhances performance in the Stroop test. **(A)** The participants were tested in the Stroop test before the training program and after 3 months of the training program. To assess inhibitory control in the Stroop test, we measured **(B)** speed-accuracy tradeoff score (*p < 0.05).

The Stroop test, a timed test of response inhibition and set-shifting, was used to test inhibitory control. The test measures cognitive functions related to attention and executive functions. This test was divided into two stages: the learning stage and the test stage. In the test, four different words (red, blue, green, and yellow) in four different colors were presented to the participants on a computer screen. The participants had to react to the color of the word and not to its meaning (Figure 4A). For example, the word red in blue color would be selected as blue. In the learning stage, 16 words were presented to the subjects without taking their score. In the test stage, 240 words were presented to the subjects. The parameters measured were: reaction time, success rate, and speed-accuracy tradeoff score.

### Statistical Analysis

Analysis of the effect of HIFT on spatial learning in the VMWM test was conducted using repeated measures (RM) two-way ANOVA in the parameters measured (latency, success, and path efficiency) for each group (control and HIFT) between the three different stages in the baseline test and after the 3-month test. In both MST and Stroop tests, RM two-way ANOVA was conducted for each group between baseline test and the 3-month time point. For success, MST, and Stroop tests, Sidak’s multiple comparisons test was used. For path efficiency and latency analysis, Tukey’s multiple comparisons test was used. Data are shown as mean ± SEM. All statistical analyses were carried out using GraphPad Prism Software.

## Results

### HIFT Enhances Short-term Spatial Learning in Adolescents

All the subjects in both groups met the inclusion criteria of participating in >90% of the training sessions. Therefore, the total number of subjects was 20 in each group. To assess spatial learning in humans, we established an experimental paradigm using the VMWM (Figure 2A). Utilizing a VR system enabled us to better mimic real-world navigation, in a manner involving spatial-learning related hippocampal and para-hippocampal cells, such as place cells, grid cells, and head direction cells. At baseline, no statistically significant difference was observed between the two experimental groups, as evident by the success rate (stage 1: 28.75% ± 6.09 vs. 35.53% ± 6.431, *p* = 0.9999, *t* = 0.748; stage 2: 40.0% ± 7.344 vs. 28.95% ± 5.495, *p* = 0.9999, *t* = 1.221; stage 3: 46.25% ± 6.091 vs. 40.79% ± 6.681, *p* = 0.9999, *t* = 0.6031; Figure 2B). Accordingly, latency to reach the target did not differ between the groups (stage 1: 29.30 s ± 1.1995 vs. 27.46 s ± 1.289, *p* = 0.9315, *t* = 1.350; stage 2: 27.33 s ± 1.1709 vs. 28.92 s ± 1.112, *p* = 0.963, *t* = 1.162; stage 3: 25.34 s ± 1.485 vs. 26.17 s ± 1.547, *p* = 0.9981, *t* = 0.6104; Figure 2C). Following 3 months of HIFT, success rate in the task improved significantly in the HIFT group (stage 1: 36.25% ± 4.959 vs. stage 3: 66.25% ± 5.524, *p* = 0.0138, *t* = 3.357, *F*_(1,38)_ = 4.049, *p* = 0.0021) compared with the control group (stage 1: 36.84% ± 7.246 vs. stage 3: 51.32% ± 5.566, *p* = 0.8424, *t* = 1.578), meanwhile following 3 months we did not found significantly differences in stage 3 when comparing between the two groups (control mean: 51.32% ± 5.566 vs. HIFT mean: 66.25% ± 5.524, *p* = 0.7957, *t* = 1.649; Figure 2B). Accordingly, following 3-month training, the HIFT group exhibited lower latency to reach the target when comparing stages 1 and 3 (stage 1: 28.46 s ± 0.888 vs. stage 3: 21.72 s ± 1.256, *p* = 0.0065, *t* = 5.001, *F*_(1,38)_ = 3.982, *p* = 0.0023; Figure 2C). The control group failed to show similar improvement (stage 1: 26.43 s ± 1.644 vs. stage 3: 25.61 s ± 1.429, *p* = 0.9984, *t* = 0.5908; Figure 2C), as in the success we did not find significant differences in stage 3 when comparing between the two groups (control mean: 25.61 s ± 1.429 vs. HIFT mean: 21.72 s ± 1.256, *p* = 0.3378, *t* = 2.848). Path efficiency is described as the Euclidean distance between the starting point and the final point divided by the total distance. Analyzing path efficiency, we found that following the 3-months training, the HIFT group exhibited a significantly higher path efficiency when comparing trial 1 to trial 4 in stage 3 (0.2615 ± 0.0424 and 0.5418 ± 0.0618, respectively; *p* = 0.0009, *t* = 5.402, *F*_(1,38)_ = 5.264, *p* = 0.0015). We did not observe a parallel significant improvement in the control group (0.1964 ± 0.0333 and 0.2644 ± 0.0517, respectively; *p* = 0.8026, *t* = 1.279; Figure 2D). Analysis of the occupation plots of the participants within the VMWM environment at baseline compared to the 3 month time point revealed that the activity of the HIFT group was more concentrated around the hidden target area compared with the control group (Figure 2E).

### HIFT Enhances Visual Pattern Separation in Adolescents

Pattern separation was assessed by calculating the LDI score, defined as the number of “similar” responses given to “lure” items minus the number of “similar” responses given to “foil” items (Figure 3A). Following 3-month training, the HIFT group exhibited significant improvement in performance in the pattern separation task, as indicated by improvements in the LDI score between baseline and following 3-month training (baseline mean: 0.2634 ± 0.0321 vs. 3-month mean: 0.3387 ± 0.0367, *p* = 0.0419, *t* = 2.407, *F*_(1,38)_ = 3.983, *p* = 0.0533) compared with controls (baseline mean 0.2517 ± 0.0299 vs. 3-month mean: 0.2658 ± 0.0543, *p* = 0.8856, *t* = 0.4411). Following 3-month we did not find significant differences comparing between the two groups (control mean: 0.2658 ± 0.0543 vs. HIFT mean: 0.3387 ± 0.0367, *p* = 0.3479; Figure 3B). In contrast with the LDI score, the HIFT group did not differ from control in recognition score (REC) defined as the number of “target” responses given to “old” items minus the number of “new” responses given to “old” items (Supplementary Figure S1).

### HIFT Enhances Inhibitory Control

The Stroop test was used to assess inhibitory control by calculating the speed-accuracy tradeoff score defined as the number of correct answers divided by the response time (Figure 4A). Following 3-month training, the HIFT group significantly improved their speed-accuracy tradeoff score from the baseline test to the second test after 3 months compared to the control group (HIFT; baseline mean: 17.19 ± 0.9506 vs. 3-month mean: 20.1 ± 1.005, *p* = 0.0066, *t* = 3.143, *F*_(1,38)_ = 13.69, *p* = 0.0007; control; baseline mean: 16.96 ± 1.1613 vs. 3-month mean: 18.96 ± 0.9837, *p* = 0.0829, *t* = 2.103). Following 3-months, we did not observe a significantly differences when comparing the two groups (control mean: 18.96 ± 0.9837 vs. HIFT mean: 20.11 ± 1.005, *p* = 0.6775; Figure 4B).

## Discussion

There is a large body of evidence for the positive effect of different types of exercise on cognitive function in animals and humans alike (Ploughman, 2008; Barak et al., 2015; Tsukamoto et al., 2016). while the combined effect of strength exercise and high-intensity aerobic training on cognitive abilities was not yet examined. In the current study, we investigated the effect of HIFT on spatial learning, visual pattern separation, and inhibitory control in adolescents. The control group performed the regular physical exercise classes. these classes differed significantly from the HIFT intervention. While the regular physical exercise classes only included moderate-intensity aerobic activities, the HIFT intervention utilized high-intensity muscle-strength exercises and “anerobic exercises.” One limitation of this study stems from the fact that each intervention group was recruited and trained at a separate school, albeit both schools exhibit identical socioeconomic state and share the same education program, reducing potential bias. The improvement in short-term spatial learning in the HIFT group is in line with studies showing that both high-intensity aerobic exercise and strength exercise improve spatial learning in rodents (Aguiar et al., 2011; Lee et al., 2012). Spatial learning is the ability to allow navigation through space, and it is correlated with enhancing neurogenesis in rodents (Lee et al., 2012; Lieberwirth et al., 2016; Tan et al., 2017). Short-term spatial learning is enhanced both in rodents and humans following exercise. This can be attributed to the highly similar experimental paradigm that was used but also to the similar cell types involved in spatial learning between humans and rodents (Barak et al., 2015).

In a study that employed the HIIT paradigm in adult humans, pattern separation abilities (as measured using the MST task) were significantly improved (Heisz et al., 2017). Adding these to our findings indicate that both HIIT and HIFT improve pattern separation. HIFT is a training method that improves both cardiovascular fitness and muscle strength (Heinrich et al., 2012; Buckley et al., 2015; Brisebois et al., 2018; Feito et al., 2018b). Given that in studies conducted in rodents, pattern separation was elevated as a result of an increase in exercise-induced neurogenesis (Creer et al., 2010; Bekinschtein et al., 2011), cardiovascular improvement following HIFT may be responsible for the effects we observed in this study on pattern separation.

Many studies have shown that aerobic exercise improves inhibitory control using the Stroop task in humans (Fortes et al., 2018; Tsuk et al., 2019). The current study shows that HIFT is also a valid training program that exerts a positive effect on inhibitory control. In elementary school and high school students, higher inhibitory control is correlated with better academic skills, math achievements, reading comprehension, self-regulation, and attention (Macleod, 1992; Tan and Holub, 2011; Fuhs and Mcneil, 2013; Kieffer et al., 2013; Oberle and Schonert-Reichl, 2013; Allan et al., 2014). Although in the current study we did not measure academic skills, we did find that HIFT improved inhibitory control. Despite the limitations of our research, we believe that these results may suggest that HIFT can be applied as an intervention for improving academic skills.

At present, it is unknown whether improvements in spatial learning and pattern separation abilities correlate with improvement in different academic skills, and additional research is needed to provide evidence for such effect. The current study, however, provides evidence that HIFT, a training paradigm that combines both strength and high-intensity aerobic exercise, significantly improves short-term spatial learning, visual pattern separation, and inhibitory control in adolescents. While we do not know specifically which part of the HIFT (strength exercise, high-intensity aerobic exercise or their combination) contributed the most to the enhanced performance in the cognitive tests we conducted, HIFT can be applied in schools because it requires a relatively short time and benefits from high adherence rates (Heinrich et al., 2014; Fisher et al., 2017).

## Data Availability Statement

All datasets presented in this study are included in the article/Supplementary Material.

## Ethics Statement

The studies involving human participants were reviewed and approved by Bar-Ilan University’s Ethics Committee in accordance with the Department of Research at the Israeli Ministry of Education. Written informed consent to participate in this study was provided by the participants’ legal guardian/next of kin.

## Author Contributions

TB-Z and EO designed the research. TB-Z and TH performed the experiments. TB-Z analyzed the results of the experiments. TB-Z and IW designed virtual reality mazes. TB-Z and TH designed inhibitory control test. MG and TB-Z designed the intervention program. TB-Z and EO wrote the manuscript.

## Conflict of Interest

The authors declare that the research was conducted in the absence of any commercial or financial relationships that could be construed as a potential conflict of interest.

## Supplementary Material

FIGURE S1 HIFT does not affect the REC score in the MST task. We measured recognition score (REC) as the number of “target” responses given to “old” items minus the number of “new” responses given to “old” items. No difference was observed between the intervention groups (*P* > 0.05).
