# Supplementary figures and images for: The Effects of High-intensity Functional Training (HIFT) on Spatial Learning, Visual Pattern Separation and Attention Span in Adolescents
Source: Front Behav Neurosci. 2020 Sep 14;14:577390. doi: 10.3389/fnbeh.2020.577390 (PMC7521200; doi:10.3389/fnbeh.2020.577390)

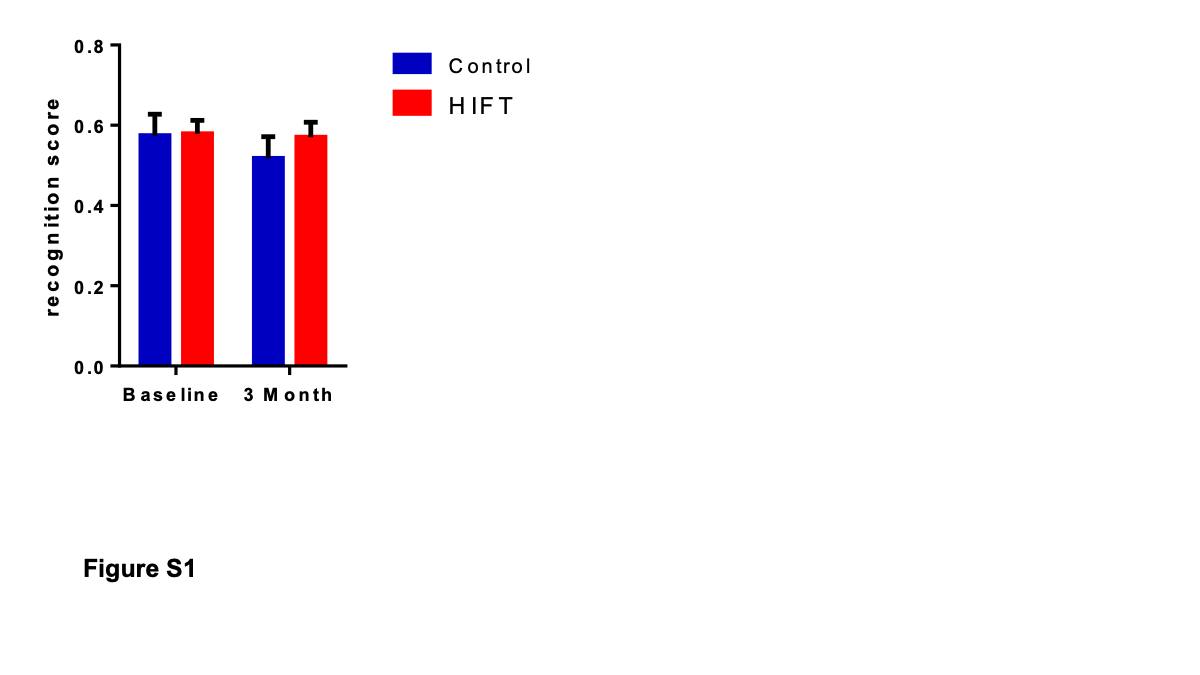

Supplement: FIGURE S1 — HIFT does not affect the REC score in the MST task. We measured recognition score (REC) as the number of “target” responses given to “old” items minus the number of “new” responses given to “old” items. No difference was observed between the intervention groups (P > 0.05). [file Image_1.TIFF]
